# Insights into Nano-Scale Physical and Mechanical Properties of Epoxy/Boehmite Nanocomposite Using Different AFM Modes

**DOI:** 10.3390/polym11020235

**Published:** 2019-02-01

**Authors:** Media Ghasem Zadeh Khorasani, Dorothee Silbernagl, Daniel Platz, Heinz Sturm

**Affiliations:** 1 Bundesanstalt für Materialforschung und -prüfung (BAM), Div. 6.6, D-12205 Berlin, Germany; dorothee.silbernagl@bam.de (D.S.); heinz.sturm@bam.de (H.S.); 2 Department Polymertechnik/Polymerphysik, Technical University of Berlin, D-10587 Berlin, Germany; 3 TU Wien, Institute of Sensor and Actuator Systems, A-1040 Vienna, Austria; Daniel.Platz@tuwien.ac.at; 4 Department Mechanical Engineering and Transport Systems, Technical University of Berlin, D-10587 Berlin, Germany

**Keywords:** nanomechanical properties, boehmite, epoxy nanocomposites, atomic force microscopy, intermodulation, interphase

## Abstract

Understanding the interaction between nanoparticles and the matrix and the properties of interphase is crucial to predict the macroscopic properties of a nanocomposite system. Here, we investigate the interaction between boehmite nanoparticles (BNPs) and epoxy using different atomic force microscopy (AFM) approaches. We demonstrate benefits of using multifrequency intermodulation AFM (ImAFM) to obtain information about conservative, dissipative and van der Waals tip-surface forces and probing local properties of nanoparticles, matrix and the interphase. We utilize scanning kelvin probe microscopy (SKPM) to probe surface potential as a tool to visualize material contrast with a physical parameter, which is independent from the mechanics of the surface. Combining the information from ImAFM stiffness and SKPM surface potential results in a precise characterization of interfacial region, demonstrating that the interphase is softer than epoxy and boehmite nanoparticles. Further, we investigated the effect of boehmite nanoparticles on the bulk properties of epoxy matrix. ImAFM stiffness maps revealed the significant stiffening effect of boehmite nanoparticles on anhydride-cured epoxy matrix. The energy dissipation of epoxy matrix locally measured by ImAFM shows a considerable increase compared to that of neat epoxy. These measurements suggest a substantial alteration of epoxy structure induced by the presence of boehmite.

## 1. Introduction

Epoxy materials are used as a matrix in carbon-fiber reinforced polymers to produce light-weight constructions for applications in such industries as automotive, aerospace and construction. Despite excellent properties such as high strength, high modulus, good adhesion, high chemical and heat resistance [1], the main challenge to overcome is the brittleness and low fracture toughness of cured epoxy matrix [2]. Among commercially available inorganic nanoparticles, boehmite nanoparticles (BNPs) have shown enhancements of mechanical properties of matrix in several polymer-based nanocomposites [3,4,5,6,7]. Particularly, BNPs show significant reinforcing effects on epoxy matrices, including increasing shear strength, shear modulus and compressive strength while improving the fracture toughness [4,8,9]. The underlying mechanism of toughening effect of BNPs on epoxy matrix is hypothesized to be due to formation of a soft interphase between epoxy and boehmite. However, the direct investigations on interphase properties of such a nanocomposite system has not yet been addressed.

The interfacial region between a filler and bulk matrix, which exhibits different chemical, physical and mechanical properties compared to bulk, is referred as interphase. It is widely accepted that the mechanical properties of composites are strongly influenced by the properties of their interphase [10]. The nature of interphase in thermoplastic and thermosetting matrices are substantially different. In thermoplastics, the interphase consists of immobilized polymer chains which exhibit less flexibility than the bulk. In thermosetting matrices however, the crosslinking chemistry at the interphase as well as in the bulk can be altered by the presence of particles. The interphase can have sizes from few nanometers up to few microns [11,12,13]. It may exhibit a property gradient or may be homogeneous [12].

Determination of interphase properties using experimental approaches is challenging due to resolution limitations in conventional mechanical characterization methods. Formation of interphases has been investigated widely in different studies using numerical methods [14,15,16,17] and or with experimental methods, for instance with temperature modulated differential scanning calorimetry (TMDSC) [18]. A direct approach to investigate mechanical properties of interphases is atomic force microscopy (AFM). AFM force–distance curve (FDC) is the most common approach to probe mechanical properties of small volumes. Especially, the ability to apply well-known models from contact mechanics (Hertz, DMT and JKR) [19] makes this method suitable for quantitative measurements of polymers. This method, has a high spatial resolution and is, therefore, suitable for probing the interphase between heterogeneous layers of material [20]. However, FDC substantially lacks the lateral resolution required to probe nano-scale domains of interphase in nanocomposites. For probing smaller volumes and resolving single nanoparticles, dynamic AFM-based approaches are required. The most common dynamic AFM mode is tapping mode which is mostly used to obtain high resolution surface topography images with additional compositional information in the tip oscillation phase image. Some studies demonstrated that the phase shift is correlated to surface stiffness [21]. However, in most cases, quantitative determination of mechanical properties is not possible with tapping mode phase image. A novel dynamic AFM technique is intermodulation AFM (ImAFM) in which a multi-frequency method provides more information about the tip-surface interaction forces than aforementioned approaches. Besides providing force curves which are equivalent to conventional FDCs, ImAFM yields information about energy dissipated by the tip-sample interaction giving insight to the viscous behavior of the material. ImAFM provides high resolution stiffness maps which makes it suitable for visualizing and for the quantitative probing of nanoscale heterogeneous phases in polymer nanocomposites. Along with stiffness maps, a second channel of information are required to distinguish the heterogenous phases (e.g., polymer and nanoparticles) and assign the mechanical properties to them. Using topography images for this purpose is not sufficiently precise particularly when the dispersed phase is too small. Moreover, mechanical approaches can be affected by topographic changes, therefore affecting the accuracy in distinguishing the border between the phases [10]. Therefore, along with ImAFM, another information channel which probes a material property independent from its mechanics, can provide higher reliability of data analysis. Scanning kelvin probe microscopy (SKPM) is commonly applied to semiconductors and conducting systems in order to determine the work function. So far, SKPM has been widely used to characterized electrical contacts, semiconductors, devices such as transistors for purposes such as determination of work function [22]. It has been also used to localize corrosion in metal alloys [23] or to measure electrical surface charges of biological samples [24]. In recent years, this method is used to probe embedded materials with different physical properties in insulating polymer matrices [25]. The electrical surface potential obtained from SKPM can be used as an information channel to visualize heterogeneous phases, even with sub-surface sensitivity [25].

In the present work, we aim to study the effect of BNPs on anhydride-cured epoxy resin. First, we focus on visualizing and mechanical characterization of interphase by combining different information channels of ImAFM together with SKPM. Second, we investigate the effect of BNPs on bulk matrix (away from particles) including stiffness, and dissipating energy. Finally, we compare the results with macroscopic mechanical analysis of these nanocomposites reported in other works and propose a describing model.

## 2. Materials and Methods

### 2.1. Materials and Sample Preparation

The epoxy system used in this study is bisphenol-A-diglycidyl ether (DGEBA, Araldite^®^ LY 556, Huntsman, Inc.) cured with an anhydride curing agent methyl tetrahydrophtalic acid anhydride (MTHPA, Aradur^®^ HY 917, Huntsman, Inc.) and accelerated by an amine, 1-methyl-imidazole (DY070, Huntsman). The mixture of epoxy, hardener and accelerator is 100:90:1 parts per weight, respectively. BNPs used in this study are commercially available spray-dried nanoparticles with orthorhombic shape and primary particle size of approx.14 nm based on the manufacturer’s datasheet (DISPERAL HP14, SASOL, Germany). First, suspensions of 30 wt % boehmite were provided and blended with DGEBA and further the hardener and accelerator are added to the blend. The concentrations used in this study is 0, 5 and 15 wt % BNP in 100:90:1 ratio of DGEBA, MTHPA and DY070, respectively. The epoxy mixture ratio used is the standard stoichiometric ratio (suggested by the manufacturer). The mixture is cured for 4 h at 80 °C to reach gelation and 4 h at 120 °C for post-curing. Dispersion and curing process was performed by Jux and coworker and described in details elsewhere [8,9]. Please note that the samples used in this study are identical to those in the above-mentioned publications. There, the reader can find more information about the dispersion and other properties in those articles, specifically that which is not mentioned in this work.

The surface of cured samples is cut with ultramicrotome to obtain a smooth surface. Before AFM measurements the surfaces of samples are ion-polished to reduce the contaminations and residues from microtome cutting.

### 2.2. Intermodulation AFM

Recently, dynamic AFM methods including usage of multi-frequency have been developed in nanomechanical studies of surfaces. In this work, we use one such multi-frequency method called Intermodulation AFM (ImAFM). In ImAFM the cantilever is excited with not only one frequency such as in tapping mode but with two frequencies close to a resonance of the cantilever. Here, we choose frequencies 0.5 kHz above and below the frequency of the first flexural eigenmode of the cantilever. Away from the surface, the cantilever performs a beating motion. Engaged to the surface, the cantilever motion is distorted by the nonlinear tip-sample interaction which creates additional frequency components in the cantilever motion spectrum as shown in Appendix A. These frequency components are called intermodulation products (IMPs), or mixing products, since they appear at frequencies which are linear integer combinations of the drive frequencies. The amplitudes and phases of IMPs are measured during scanning with a multi-frequency lock-in amplifier. At each pixel, hundreds of oscillations are carried out starting from low amplitudes, reaching a maximum and decreased to zero. As this cycle takes less than few milli-seconds, ImAFM has the advantage of being much faster, as compared to the conventional force–distance curves (FDC).

**Figure 1:**
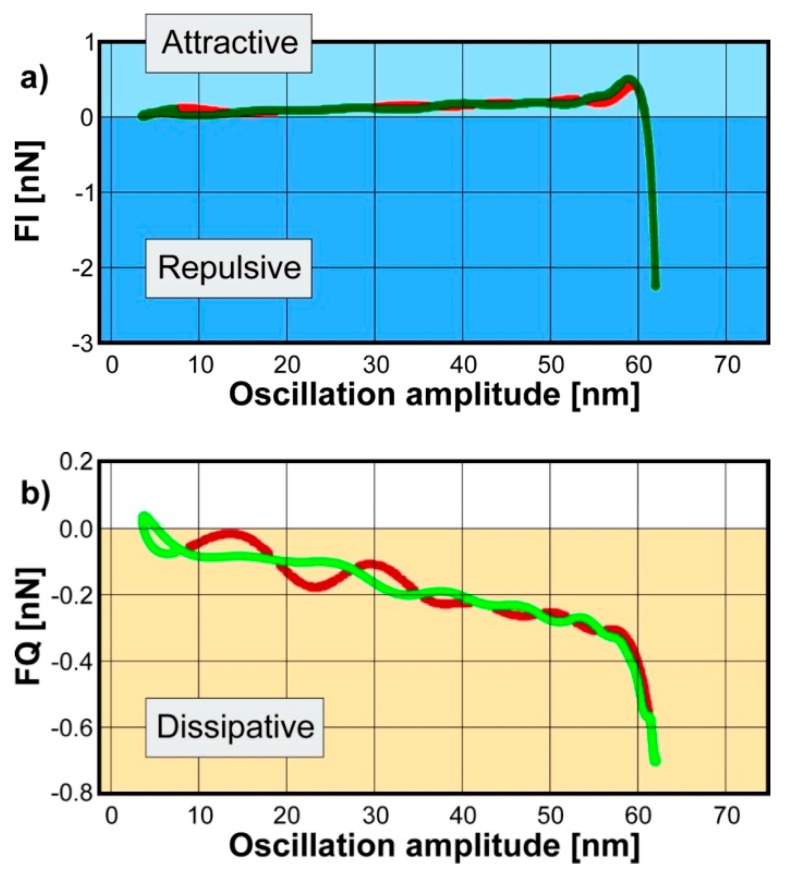
Reconstructed conservative *F*_I_ (**a**) and dissipative *F*_Q_ (**b**) forces on a polymer substrate. the red and green lines present the approach and retract curves, respectively.

The IMPs are directly correlated to the tip-surface force. For a single pixel, we can visualize this correlation with force quadrature curves which show the in-phase and out-of-phase component of the force with respect to the tip motion for each oscillation cycle [26]. The in-phase component *F*_I_ corresponds to the conservative part of the force describing the elastic behavior of the surface. The out-of-phase quadrature *F*_Q_ measures the dissipated energy during a single oscillation cycle. Examples of *F*_I_ and *F*_Q_ curves are presented in Figure 1. *F*_I_*(A)* looks similar to those conventional force–distance curves: it consists of an attractive and repulsive regime. The amplitude in the beginning of the measuring cycle is low therefore there is no tip-surface interaction. By increasing the amplitude, tip and sample spends more time closer and the tip gets into attractive regime (positive values of *F*_I_) which is due to van der Waals forces. With further increase of the amplitude, the tip makes contact with the surface and penetrates into it. In this region the tip experiences a net repulsive force (negative values of *F*_I_). However, at this stage *F*_I_*(A)* cannot be treated directly as FDC curves since the force is plotted as a function of oscillation amplitude rather than tip position. Amplitude-dependence force spectroscopy (ADFS) uses the inverse Abel transform to converts *F*_I_*(A)* to a traditional force-tip position curves [27,28]. The ADFS curves can be treated as FDC curves: the slope of the curve in the repulsive regime gives a quantitative measure of the stiffness describing the purely elastic responds of the measured sample volume. The force in attractive regime mainly originates from van der Waals forces which are caused by dipole–dipole and dipole-induced dipole interactions between the tip and surface. Therefore, the work of attractive forces includes information about material changes which is independent from the surface mechanics.

The *F*_Q_*(A)* describes the dissipative part of tip-surface interaction, which originates from viscous nature of the material [29].

For the analysis of spatially varying features, we create surface maps of the ADFS stiffness, the attractive force and the total energy dissipated during in a single pixel. It is noteworthy that the force quadrature curves shown in Figure 1 are measurements of single pixels whereas the maps show the measurement of a complete surface. Details about calculation of energy dissipation from multifrequency data can be found in Appendix B.

ImAFM measurements were carried out using MFP3D microscope (Asylum Research, Santa Barbara, CA, USA). A multi-frequency lock-in amplifier (Intermodulation Products, Segersta, Sweden) is used to generate the drive signals and measure the intermodulation spectra. The probes are HQ:NSC35 (Mikromasch, Wetzlar, Germany) with resonance frequency of 190 kHz (for measurements shown in Section 3.1) and 202 kHz (for measurements shown in Section 3.2), with tip radius lower than 20 nm.

### 2.3. Scanning Kelvin Probe Microscopy

The vibrating capacitor or kelvin probe is a method to measure the contact potential difference (CPD) between a sample and tip also called surface potential *V*_sp_ [30]. The sample and probe behave as a capacitor plate with air as the dielectric in between. *V*_sp_ depends mainly on difference between work functions of probe and the sample. To obtain high lateral resolution surface potential maps, scanning kelvin probe microscopy (SKPM) is used. In this method, an AC signal excites the cantilever electrostatically at its resonance frequency. The potential difference between probe and the surface results in the mechanical oscillation of cantilever. The feedback loop nulls the oscillation by applying a bias voltage to the cantilever. This bias voltage is then collected as a contact potential difference (CPD). The corresponding equations and technical considerations are described in detail elsewhere [31]. SKPM is usually carried out as a dual-pass approach, performing two scans per line on the selected scan area. The first pass which includes the mechanical excitation of the cantilever (tapping mode) obtains the topography of the line. In the second pass, which is known as lift or nap mode, the topography information is used to maintain a defined distance from the surface which is known as nap height. Choosing a suitable nap height is crucial for increasing the resolution of SKPM while avoiding touching the surface during the second pass.

In this work we used MFP3D microscope (Asylum Research, Santa Barbara, CA, USA) in SKPM mode. The gold-coated silicon probes with resonance frequency of 190.130 kHz, radius lower than 20 nm provided by Mikromasch (Wetzlar, Germany) was used. During all SKPM measurements nap height is chosen to be 50 nm as the suitable height according to topographic features of the surface. The resulted scans shown in this article are corrected by offset plane with the purpose of enhancing the visibility of the contrast. Therefore, the scale shown in SKPM images are different than the actual values. The measurements were carried out in air, at room temperature, using the first eigenmode frequency. Therefore no major subsurface sensitivity is expected, since this would mainly be the case when using the second eigenmode [32].

## 3. Results 

In Section 3.1, we focus on distinguishing particles, visualization of the interphase and determination of its stiffness. We use an epoxy/boehmite nanocomposite with 5 wt % nanoparticles (EP/BNP5) as this weight percentage is high enough to show mechanical improvements in the macroscale meanwhile not so high that the particle agglomerations become dominant over the scanned surface [9]. We obtain the ImAFM stiffness and attractive forces of different phases of the nanocomposite including particles, interphase and matrix, meanwhile using potential map obtained by the SKPM mode as a complementary tool to verify the presence of particles.

In Section 3.2, we quantify the effect of nanoparticles on the bulk matrix. The stiffness, work of attractive forces, and energy dissipation of the matrix phase in a high concentration nanocomposite with 15 wt % BNPs are derived from ImAFM measurements and compared to those of neat epoxy. 15 wt % concentration was specifically chosen since this nanocomposite has the highest Young’s modulus among other concentrations measured in our previous study, meanwhile the epoxy matrix in this nanocomposite possesses the lowest crosslinking density [33]. Hence, it is hypothesized that with this filler concentration the properties of the epoxy matrix are strongly altered by BNPs.

### 3.1. ImAFM and SKPM Studies on Epoxy with 5 wt % BNP 

**Figure 2:**
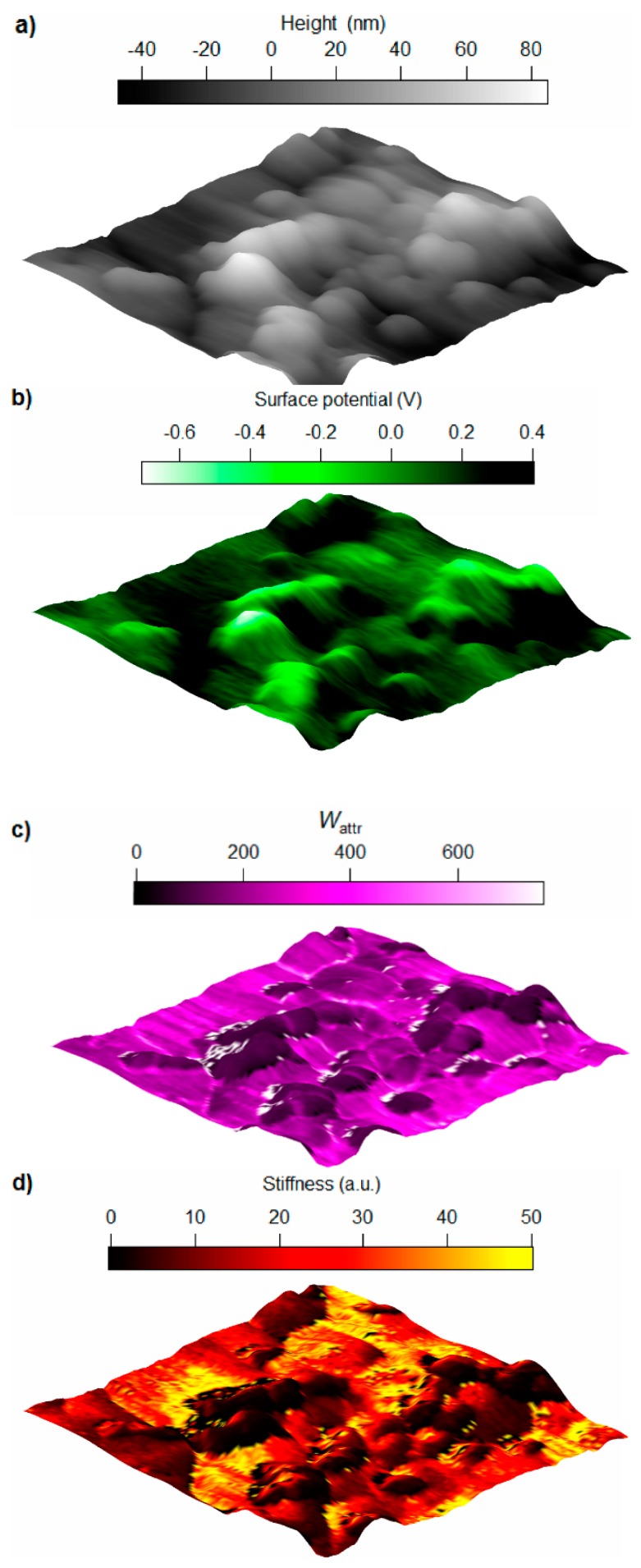
(**a**) The 3-dimensional tapping mode topography; (**b**) Surface potential; (**c**) Work of attractive forces *W*_attr_ and (**d**) stiffness maps of epoxy/boehmite nanocomposite with 5 wt % nanoparticles. The scan sizes in all images are 860 nm × 860 nm. White pixels in *W*_attr_ show error.

Figure 2 shows AFM data acquired from a region located on the surface of EP/BNP5. The overview of a larger scan area is provided in Appendix C. The topography image (Figure 2a) shows protrusions with different sizes. The main challenge is to distinguish the features related to presences of BNPs from nodular structures which are commonly observed in cured epoxy systems [34]. For this purpose, potential map obtained by the SKPM mode is used as a complementary tool to verify the presence of BNPs. Generally speaking, the potential values are related to the work function and electronic state of the surface which is actually a signal to measure the material contrast [24]. In Figure 2b, the surface potential map shows contrast between the protrusions and the rest of the surface which verifies the presence of BNPs within these areas. Please note that in most conductive cantilevers, the entire bottom side of cantilever is coated with a conductive layer (here, gold). Therefore, the signal is not limited to the capacitance formed between the tip apex and the sample, but the entire cone is participating in producing the signal. Despite such limitations in the lateral resolution, SKPM clearly identifies compositional contrasts with the precision required in this work.

Figure 2c,d show the work of attractive forces *W*_attr_ and stiffness maps, respectively, generated from ADFS curves. In a single ADFS curve obtained for each pixel, *W*_attr_ is calculated from the net attractive regime and the slope of the curve is proportional to stiffness. The *W*_attr_ map shows a clear contrast between the protrusions and the surrounding with a well-defined border. The area with sudden decrease in *W*_attr_ is located where the potential maps shows the presence of boehmite. Considering the van der Waals forces as the main driving force for net attractive regime, the low values of *W*_attr_ is an indication for a weaker van der Waals forces between the tip (gold) and BNPs than the epoxy. Van der Waals forces which are mainly originated from dipole–dipole and dipole-induced dipole interactions between tip and the surface, can be used as an additional information channel about the surface composition independent from its mechanics. Thus, when measuring the mechanical response with ImAFM, *W*_attr_ signal can also be used to visualize material contrast.

Despite the existence of two distinguishable phases in Figure 2c, the contrast in stiffness map (Figure 4) shows a variety of stiffness values in different distances from the protrusions. The area related to protrusions shows two phases, an area close to the center of protrusions with higher stiffness surrounded by an extremely low stiffness area (shown in black color). It is noteworthy that the soft area is located at an immediate distance from nanoparticles located by potential and *W*_attr_ maps. Therefore, the soft area relates to the particle-polymer interphase.

It is noteworthy that the bulk matrix shows variations in stiffness in the scanned area. Blocks with high (yellow) and low (red) stiffnesses in bulk epoxy indicate the inhomogeneous nature of the matrix.

Since sudden height changes affect the force measurements, it is crucial to investigate the roll of topography artifacts. Detailed analysis of topography-stiffness relation for the scanned surface is presented in Appendix D. This analysis demonstrates that except minor points with sudden changes of height and groove-like topographic features, most of topography changes and the stiffness values are independent from each other. Thus, by excluding the affected points of the scanned areas as error points, the remaining ADFS curves are independent from topography artifacts.

**Figure 3:**
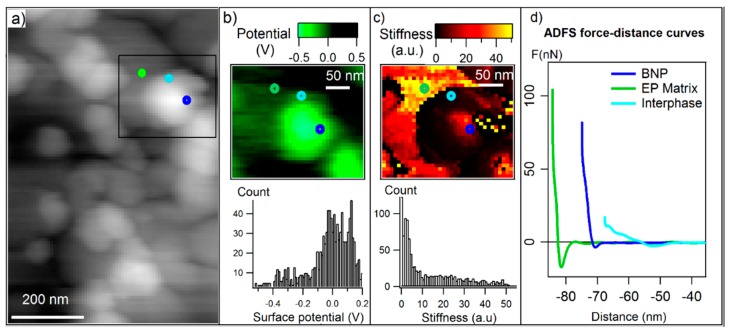
(**a**) 9AFM tapping mode topography with the selected region of analysis marked with a square box; (**b**) surface potential map and histogram and (**c**) amplitude-dependence force spectroscopy (ADFS) stiffness map and histogram of the selected area; (**d**) ADFS curves related to three points shown with circle markers on the maps with the approximation of the location of boehmite nanoparticles (BNPs) (dark blue), epoxy matrix (green) and interphase (light blue).

**Figure 4:**
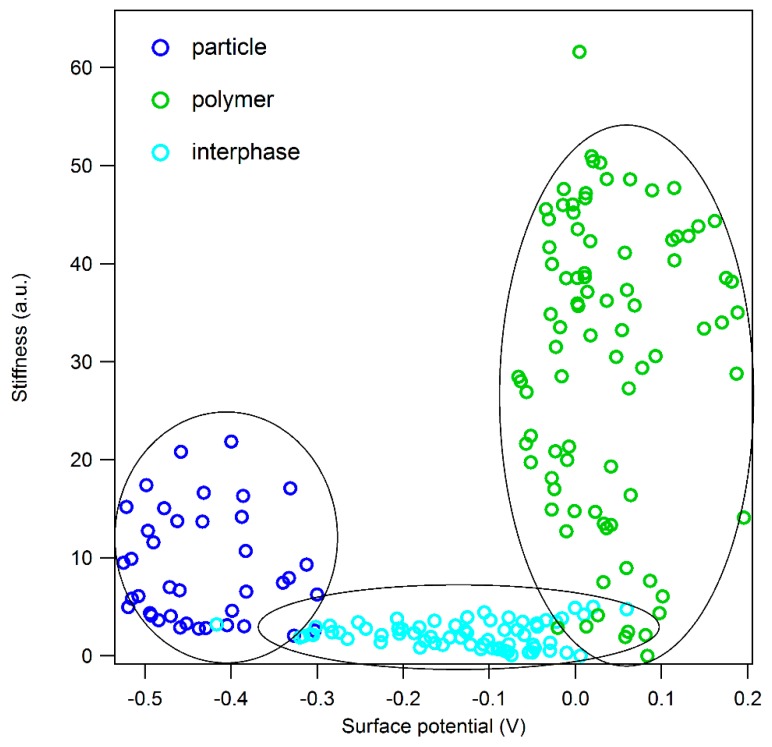
Two-dimensional histogram of stiffness vs. surface potential of the selected area (shown in Figure 3) of the scanned surface of EP/BNP 5. The dashed lines are used to help the eyes to distinguish between three different regions of the histogram.

To precisely distinguish the stiffness of particles, interphase and polymer, several areas with the presence of nanoparticles are selected and analyzed separately (Appendix E). One of the selected areas is shown in the topography image (Figure 3a), and the corresponding maps of surface potential and stiffness are presented in Figure 3b,c, respectively. The surface potential distinguishes the nanoparticles from matrix however the interfacial region is not resolved in the potential map. Meanwhile, in the stiffness map, the existences of a soft region in the vicinity of particles is clearly observable. In Figure 3d, single ADFS curves of selected points with different distances from the particle are presented compared. Here, it is also shown that the stiffness (slope) of the point in the interfacial region is drastically low. To precisely relate all the measured stiffness in the scanned area to different phase of the nanocomposite (particle, matrix and interphase), we use the material contrast shown in surface potential map together with the stiffness map. For this purpose, we combine two information channels of stiffness and surface potential—and plot a two-dimensional histogram as shown in Figure 4. It is noteworthy that the measured points which were affected by sudden topographic changes are considered as error and excluded from the 2D histogram cloud.

In the 2D histogram, the stiffness values are sorted based on the corresponding surface potential values and three distinguishable regions (marked with dashed circle lines in Figure 4) are clearly observable on the histogram cloud. In the following, each region is discussed separately:

(1) Dark blue points are related to pure BNP particles as they exhibit negative surface potential values (from −0.3 V to −0.5 V). They have large distributions of stiffness varying from 5 up to 22. The variation of stiffness values in the area related to pure BNPs can be due to following reasons: (i) Due to anisotropic nature of boehmite crystals, force curves obtained from different orientations show different stiffness values. (ii) Particles which are present in the nanocomposites are in fact secondary particles which are formed by aggregation of several primary particles with the size of 14 nms. Therefore, while in contact with the tip, several intra and inter-slippage between layers can occur which helps the deformation and results in apparent stiffness values which may be lower than the actual values.
(2) Green points are related to the pure matrix, far from the particle, according to their surface potential values. In this area, potential values are mostly positive and have a narrower distribution (between −0.05 and 0.2 V) compared to that of BNPs. The stiffness variation in epoxy matrix is high the values are distributed between 5 to 50. The broad distribution of stiffness in epoxy phase is due to following reasons: i) Inhomogeneous phases in epoxy-anhydride cured systems which has been already reported in several studies [35,36]. ii) Local changes in stoichiometric ratio which results in changes in the chemical structure of the network density and thus affect the mechanical properties of the epoxy [33].
(3) The light blue cloud is related to the matrix in the immediate proximity of particles. This interfacial region has a gradient potential, but no gradient in stiffness is observed. The potential values start from low values in vicinity of particles (−0.3 V) increasing up to 0.05 V when getting close to the pure matrix. In all distances from the particle, the interphase shows stiffness values between 1 to 5. The homogenous interphase is unlike commonly reported interphase formation in which there was a gradient in property changes were observed [12]. The soft interphase appears as a phase segregation which can be due to several effects. One is the preferential absorption of one of epoxy components (DGEBA monomers or anhydride curing agents) on the surface of BNPs. This hypothesis is discussed further in Section 4.

One surprising observation is the average stiffness of BNP particles which is lower than that of epoxy phase. Contrary to the structural stiffness of boehmite calculated by simulation which suggest a modulus value between 136 and 267 GPa with respect to plane orientation, Fankhänel and coworkers reported an experimental average modulus of 10 GPa [37]. This behavior is suggested to be due to the slippage behavior between the layers and weak interlayer bonding. Nevertheless, knowing that the neat anhydride-cured epoxy has a Young’s modulus of approx. 3.3 GPa [4,38], it is expected that in our nanocomposite system, particles exhibit higher stiffness values than the polymer matrix. To understand this unexpected inversion of stiffness between filler and matrix, in the next section, properties of the matrix phase of EP/BNP nanocomposites are investigated and compared with neat cured-epoxy.

### 3.2. ImAFM Studies on Neat Epoxy and Epoxy with 15 wt % BNP

**Figure 5:**
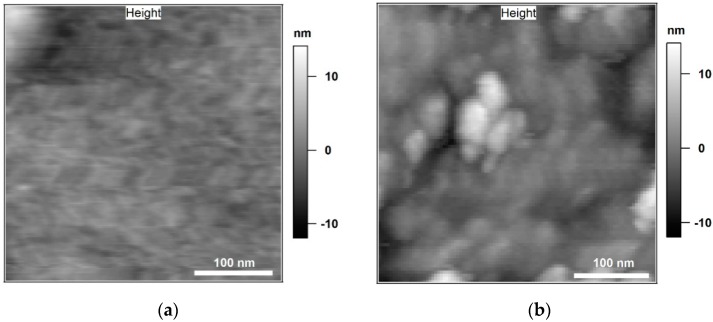
Tapping mode topography of 350 nm × 350 nm scan area of neat epoxy (**a**) and epoxy with 15 wt % BNPs (EP/BNP15) (**b**).

In Figure 5 topography images of neat cured epoxy and epoxy/BNP nanocomposite with 15 wt % particle content (EP/BNP15), respectively are compared. Moreover, an overview of the particle distribution in EP/BNP15 is provided by operating scanning electron microscopy in transmission mode (Appendix F). Although larger agglomerates were scarcely observed, the majority of surface contains particles in form of agglomerates with the size of less than 100 nm similar to what is observed in Figure 5b. The area shown here in Figure 5b was carefully selected so as to avoid large agglomerates.

**Figure 6:**
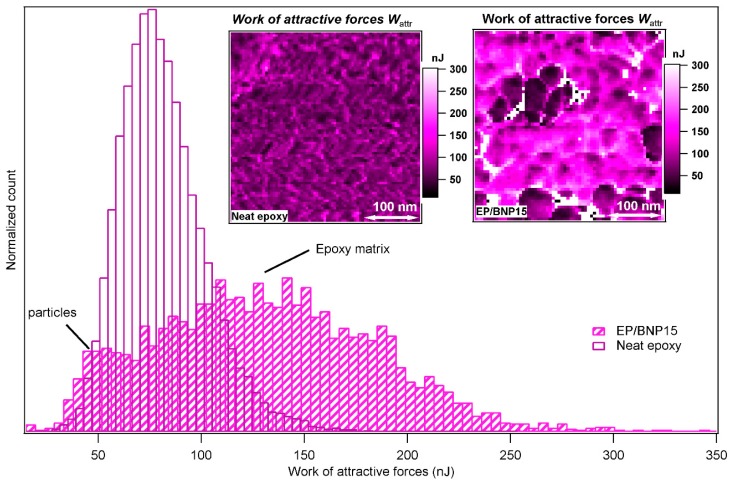
The comparison of the histograms of work of attractive forces *W*_attr_ in neat epoxy and EP/BNP15. The left-side inset image is *W*_attr_ map of neat epoxy and the right-side is *W*_attr_ map of EP/BNP15.

In Figure 6, the *W*_attr_ of neat epoxy and EP/BNP15 are compared. As previously discussed, *W*_attr_ data channel shows contrast between BNPs and epoxy phase independent from the mechanical properties. Here as well, in the inset image of Figure 5, the *W*_attr_ image of EP/BNP15 shows contrast between BNP and polymer phase. The *W*_attr_ of neat epoxy also contains inhomogeneities which is due to well-known nodular structures of epoxy. Nevertheless, the surprising observation is that in EP/BNP15, the pure matrix phase in presence of BNPs has higher values of *W*_attr_ compared to neat epoxy. The comparison of *W*_attr_ histograms shown in Figure 5 demonstrates 100% increase in *W*_attr_ for the matrix. This is a clear indication that BNPs induces physical/chemical alteration in epoxy which is worthwhile for further investigations.

**Figure 7:**
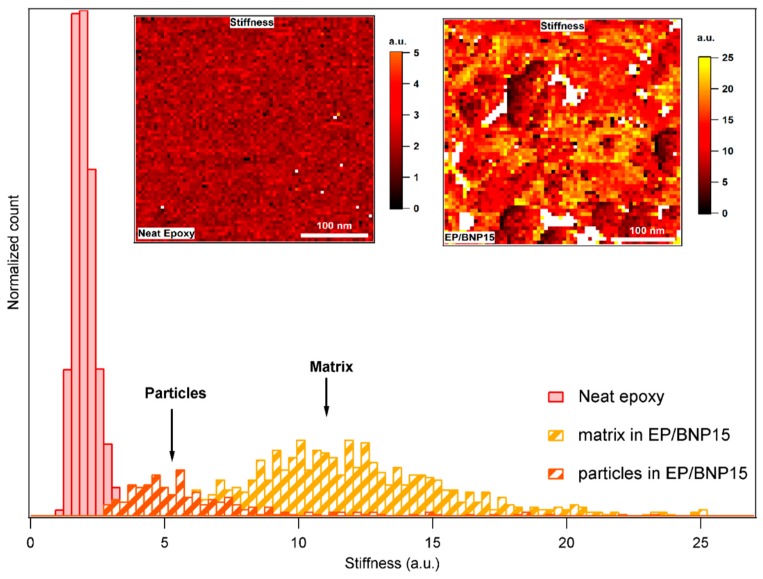
Comparison of stiffness histograms of neat epoxy and EP/BNP15. The left-side inset image related to stiffness map of neat epoxy and the right-side to EP/BNP15.

Figure 7 compares the stiffness of neat epoxy with epoxy matrix in EP/BNP15. The histograms show that the stiffnesses of nanoparticles are slightly higher than of neat epoxy, as expected. However, this relationship is inversed in EP/BNP15 in which matrix is stiffer than the particles. The inversed situation with particles softer than the epoxy matrix has been also observed in Section 3.1. The comparison between the stiffness of matrix in EP/BNP15 and neat epoxy reveals a 100% to 400% increase in matrix stiffness which occurs in the presence of boehmite. This is a significant change property of epoxy.

**Figure 8:**
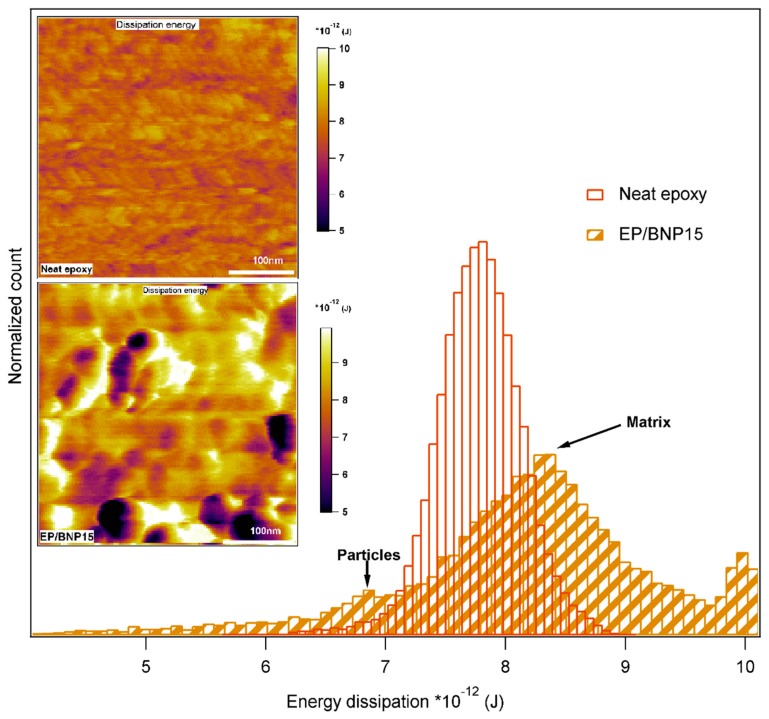
Comparison of energy dissipation histograms in neat epoxy and EP/BNP15. The top inset image related to energy dissipation map of neat epoxy and the bottom image to EP/BNP15.

Figure 8 compares the energy dissipation maps of neat epoxy with EP/BNP15. It is observed that in EP/BNP15, the energy dissipation of particles is lower than of epoxy matrix. Clearly, long chains of polymer can dissipate the energy more than BNPs with crystal structures. However, comparing the peak values of energy dissipation histograms, it is observed that epoxy matrix in EP/BNP15 shows an approx. 10% increase of energy dissipation compared to neat epoxy. This also indicates physical alteration of epoxy matrix as an effect of boehmite nanoparticles.

## 4. Discussion

The analysis of ADFS curves in EP/BNP5 presented in Section 3.1 demonstrated the formation of an interfacial region which has a significantly low stiffness. This region appears mostly as a block of homogeneously soft material in the vicinity of particles rather than a region with stiffness gradient. As we have reported elsewhere, BNPs induce changes in epoxy matrix which result in different thermomechanical behavior and a significant decrease of crosslinking density [33]. One hypothesis is that a disturbed crosslink density near the particles results in formation of a soft interphase. However, it has been demonstrated in several works on epoxy systems that the glassy state modulus does not reflect the crosslinking properties of the material, but exhibits the noncovalent bonding and inter and intra-molecular packing [39,40]. It was demonstrated that a low crosslinking density system can exhibit higher modulus at glassy state. Therefore, the disturbed crosslink density cannot explain the formation of a soft interphase. Another hypothesis is the accumulation of one component of epoxy mixture (either DGEBA or anhydride hardener or both) on the surface of particles due to preferential absorption, covalent or noncovalent bonding with boehmite, leading to a local phase segregation. This effect was observed in an epoxy-copper layered composite that a hard interphase was formed due to formation of regions with different amount of amine hardener as an effect of preferential absorption of the copper layer [12]. In our case, the local stoichiometric ratio of epoxy and hardener can also vary in bulk matrix and thus resulting in alteration of chemical and physical properties of the matrix. For further investigations on the chemical composition of the interphase we use high resolution infrared-AFM in order to verify this hypothesis.

In Section 3.2, the effect of BNPs on epoxy matrix was investigated. The significant increase in stiffness, attractive forces and energy dissipation in bulk matrix compared to neat epoxy demonstrated that boehmite induces physical, mechanical and chemical property alteration in anhydride-cured epoxy matrix. It was demonstrated that these property alterations in epoxy are not only limited to the interfacial region, but the bulk epoxy is affected significantly. The changes in epoxy matrix can affect the macroscopic properties of the composite significantly, even more so than more than what the interphase can do. Therefore, when applying models, such as Halpin–Tsai [41,42], also the increased modulus of the matrix must be taken into account. It is noteworthy that the stiffening effect of nanoparticles in crosslinked matrices has already been reported. Using the ImAFM approach, they observed an increase of stiffness in PDMS matrix in the presence of silica nanoparticles.

We were also able to show that, although BNPs themselves exhibit a lower energy dissipation than polymer matrix (as seen in Figure 7), they induce changes in the matrix structure which result in the increase of energy dissipation in bulk polymer. The fracture toughness and critical energy release rate increase in epoxy-boehmite nanocomposites which was reported previously verifies this observation [9].

## 5. Conclusions

In this article, we applied different AFM-based methods to visualize property contrast and probe mechanical properties of nanoparticles, polymer matrix and the interphase in epoxy-boehmite nanocomposite systems. Multi-frequency intermodulation AFM (ImAFM) was used as a tool to measure forces together with scanning kelvin probe macroscopy (SKPM) as an additional information channel to show material contrast independent from the mechanics of the surface. ImAFM maps demonstrated stiffness contrast between polymer, particle and the interphase. SKPM shows potential contrast between boehmite nanoparticles and epoxy matrix. Combination of mechanical and surface potential values led to a more precise determination of the location and stiffness of interphase. The results demonstrated the presence of a soft block of polymer near the interfacial region with no visible stiffness gradient. The stiffness of this region is considerably lower than both particles and polymer phase.

Moreover, the effect of boehmite on the matrix properties was investigated by focusing on stiffness and energy dissipation during the tip-surface interaction obtained from ImAFM force curves. A significant stiffening effect of boehmite nanoparticles on anhydride-cured DGEBA was demonstrated. Meanwhile, the presence of boehmite resulted in increase of energy dissipation. We suggest that boehmite cause structural alteration of matrix by inducing local changes in stoichiometric ratio of the epoxy and hardener due to preferential surface absorption, covalent or non-covalent bonding between boehmite particles and mixture components.

## Figures and Tables

**Figure A1:**
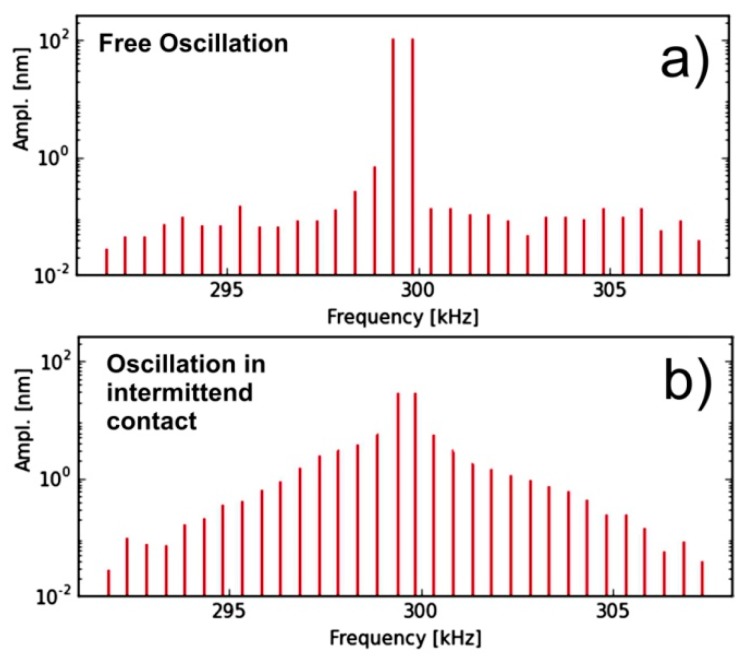
Free oscillation (**a**) and oscillation in intermittent contact (**b**) of a cantilever with *ω*_0_ = 299.6 kHz on a polymer surface.

**Figure A2:**
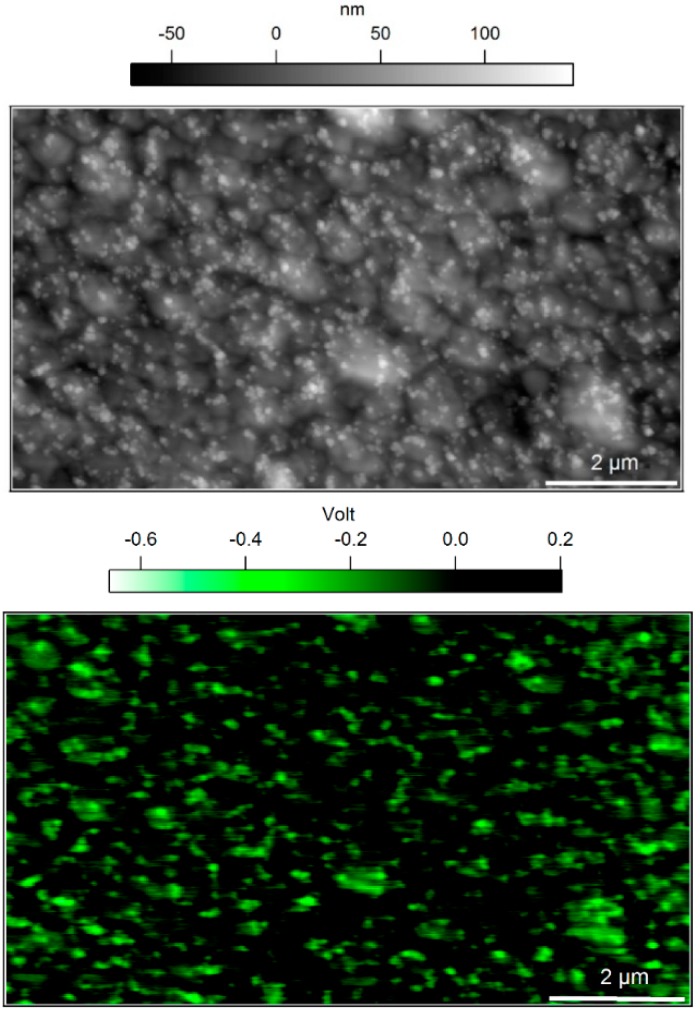
Tapping mode topography (**top**) and SKPM surface potential map (**bottom**) of a 10 µm × 6 µm scan area of EP/BNP5.

**Figure A3:**
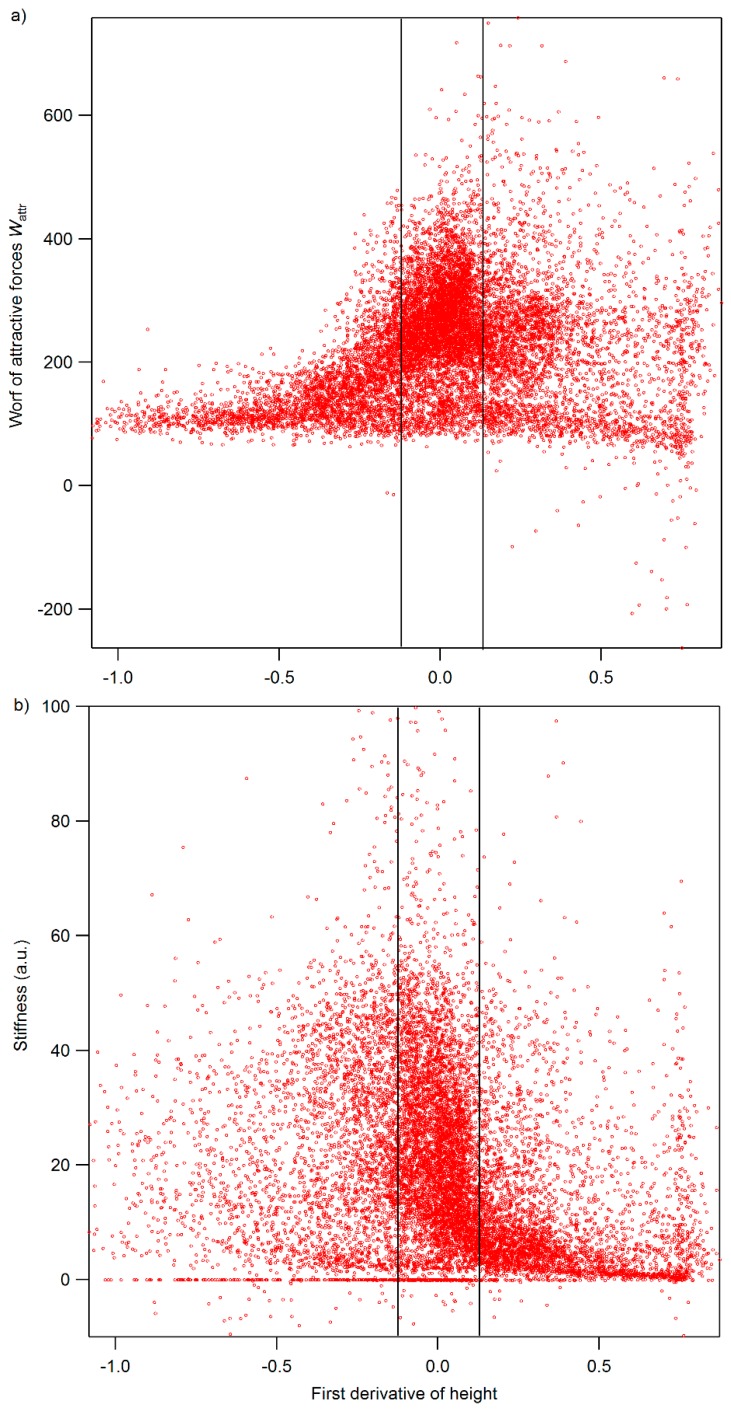
The effect of topography changes on ImAFM force measurement. (**a**) histogram of ImAFM stiffness and (**b**) work of attractive force *W*_attr_ versus the histogram first derivative of height obtained from tapping mode.

**Figure A4:**
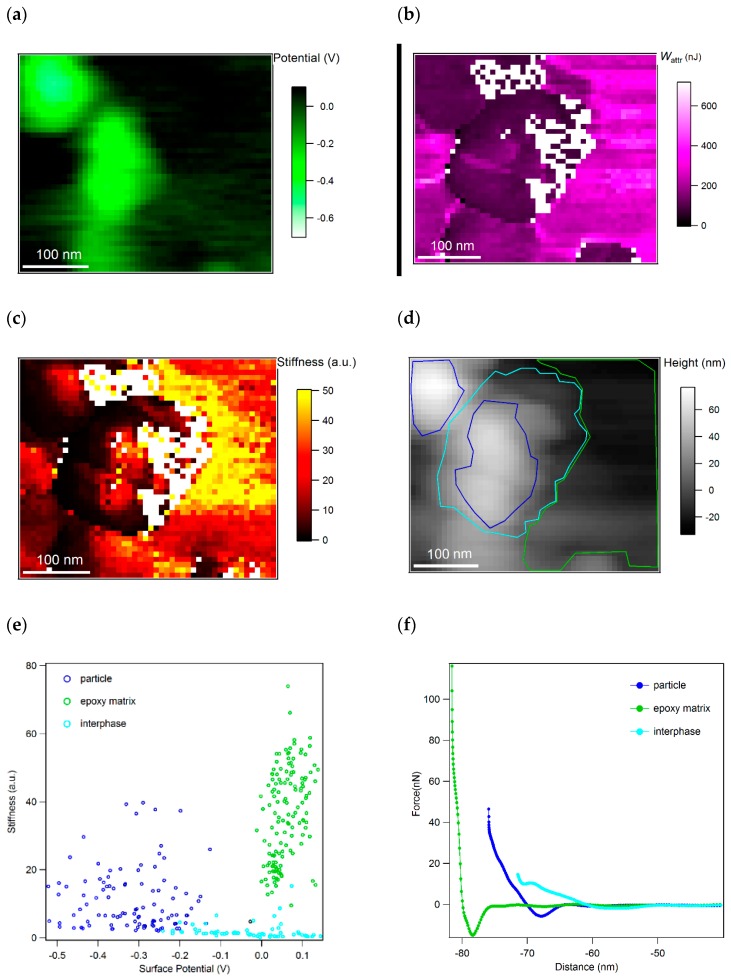
(**a**) Surface potential; (**b**) work of attractive forces *W*_attr_ and (**c**) the stiffness map of a selected area from Figure 2 scans. (**d**) The corresponding topography of the scanned area where the domains of particle, interphase and pure matrix are distinguished by traces with dark blue, light blue and green color, respectively. (**e**) 2D histogram of stiffness vs. potential. (**f**) typical ADFS curves related to particles (dark blue), interphase (light blue) and pure matrix(green). White pixels in (**b**,**c**) indicate error pixels.

**Figure A5:**
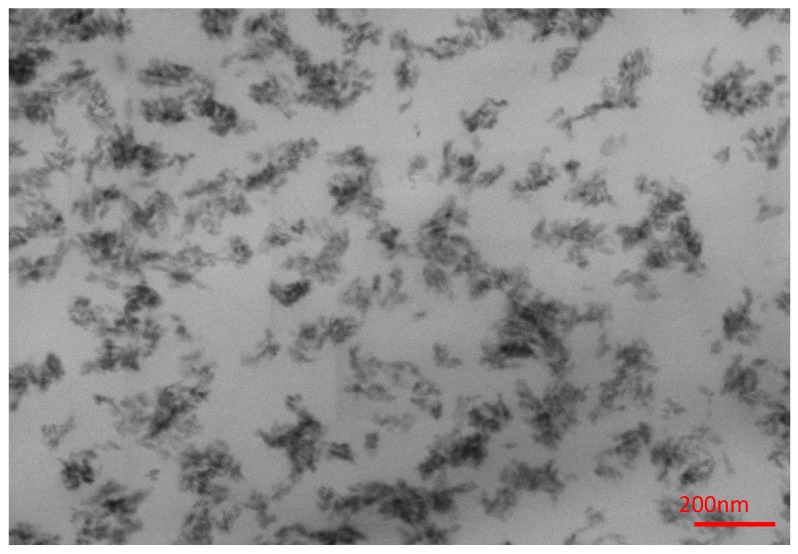
T-SEM micrograph of a 100 nm thick microtome cut of the EP/BNPT5.

## Appendix B

Energy dissipation in multifrequency AFM:

In conventional dynamic atomic force microscopy (AFM) a sharp tip at the end of a micro-cantilever oscillates sinusoidally close to a surface of a sample. The phase difference between the tip oscillation and the drive force or the tip motion far away from the surface is can be related to the energy or power dissipated by the tip-surface interaction. Thus, the (oscillation) phase signal is often considered as a valuable channel of information giving compositional contrast between different surface materials.

Recently, a variety of multifrequency AFM methods have been developed giving detailed insight into the tip-surface interaction. However, the in multifrequency AFM the tip motion is no longer purely sinusoidal and thus the equations relating dissipated energy and phase signal in conventional dynamic AFM do not hold for multifrequency AFM methods such as Intermodulation AFM. Here, we show how similar relations can be derived for general multifrequency techniques allowing for simple computation of the energy dissipated directly from the measured tip motion spectrum. Generally, the energy *E_dis_* dissipated by the tip-surface force *F_ts_* is given by the integral

(A1) $$E_{dis}=\int_{C}F_{ts}\left(z\right)dz$$

where *C* is the trajectory of the tip in the *z* coordinate. This expression can be written as a parametric integral,

(A2) $$E_{dis}=\int_{C}^{T}F_{ts}\left(t\right)\dot{z}dt$$

where *T* is the period of the tip motion and the dot denotes derivation with respect to time *t*. We introduce the time-reversed velocity which is defined as $\dot{z}-t=\dot{z}\left(-t\right)$ which yields

(A3) $$E_{dis}=\int_{C}^{T}F_{ts}\left(t\right)\dot{z}-\left(0-t\right)dt$$

This integral can be identified as a convolution and allows by the Fourier convolution theorem us to establish a relation between the dissipated energy *E_dis_* and the spectrum of the tip motion,

(A4) $$E_{dis}=\;{Ғ}^{-1}\{-i\omega {\hat{F}}_{ts}.\;{\hat{z}}^{*}\}\left(0\right)$$

where Ғ is the Fourier operator, *i* is the complex unit, *ω* is the frequency variable, the hat denotes a Fourier transformed quantity and the star the complex conjugate. In the last step we have used that the Fourier transform of the time-reversed velocity can be expressed as

(A5) $$\dot{z}-\left(\omega \right)=-i\omega {\hat{z}}^{*}\left(\omega \right)$$

With the cantilever transfer function $\hat{G}$, we can determine the spectrum of the tip-surface force ${\hat{F}}_{ts}$ from the measured tip-motion spectrum close to the surface $\hat{z}$ and far away from the surface ${\hat{z}}_{free}$,

(A6) $${\hat{F}}_{ts}={\hat{G}}^{-1}\left(\hat{z}-{\hat{z}}_{0}\right)$$

In case of a linear single mode cantilever, the transfer function is given by

(A7) $$\hat{G}\left(\omega \right)=\frac{\omega_{0}^{2}/k}{\omega_{0}^{2}-\omega^{2}+i\frac{\omega_{0}\omega }{Q}}$$

where $\omega_{0}$ is the angular resonance frequency, *k* is the spring constant and *Q* is the quality factor of the cantilever. The dissipated energy *E_dis_* now becomes

(A8) $$E_{dis}={Ғ}^{-1}\{-i\omega \hat{G}\left|\hat{z}\right|{}^{2}\}\left(0\right)-{Ғ}^{-1}\{-i\omega \hat{G}{\hat{z}}_{0}{\hat{z}}^{*}\}\left(0\right)$$

With this relation we can determine the energy dissipated by the tip-surface force during one period of the tip motion directly from the measured free and engaged tip motion spectra.

## Appendix C. Overview of Nanoparticle Distribution in EP/BNP5

Based on the contrasts observed in Figure A2, particles tend to form agglomerates in the average size 100 nms. since the primary particle size is 14 nm, what we refer here as particles as actually secondary particles in the form of aggregation of few 10 primary particles.

## Appendix D

### Analysis of Topography Artifacts in Measured Stiffness

Sudden height changes can cause artefacts in topography and force measurements, known convolution effect [43]. Here we study the effect of topography changes on force measurements including stiffness and attractive forces. The first derivative of the topography is calculated and plotted over the force measurement values. Figure A3 shows the histogram of stiffness and work of attractive forces versus the histogram of topography (first derivative). For both highly negative and positive values of height derivative which indicates areas of with extreme up and downhills in topography, *W*_attr_ shows low values, however in flat areas (where the derivative is zero or close to zero) *W*_attr_ exhibit both low and high values. It can be concluded that expect the highly extreme topography changes, the values of *W*_attr_ are not affected by topography changes. The same can be observed for stiffness: Zero values of stiffness which we take as error occur all over the surface from flat to sharply angled. Also, in flat areas the stiffness can vary from 0 to 100.

Topography features rarely cause artifacts in Potential measurement as in SKPM the tip is kept far from the surface and does not come into contact with the surface.

## Appendix E

Analysis of stiffness-potential relationship of a selected area.
